# Photo-catalytic Killing of HeLa Cancer Cells Using Facile Synthesized Pure and Ag Loaded WO_3_ Nanoparticles

**DOI:** 10.1038/s41598-018-33434-7

**Published:** 2018-10-15

**Authors:** Rasha A. AbuMousa, Umair Baig, Mohammed A. Gondal, Mohamad S. AlSalhi, Fulwah Yahya Alqahtani, Sultan Akhtar, Fadilah Sfouq Aleanizy, Mohamed A. Dastageer

**Affiliations:** 1 Department of Physics & Astronomy, College of Science, King Saud University, Riyadh, 11451 Saudi Arabia; 2 Center for Research Excellence in Desalination and water Treatment, King Fahd University of Petroleum & Minerals, Dhahran, 31261 Saudi Arabia; 3 Laser Research Group, Physics Department & Center of Excellence in Nanotechnology, King Fahd University of Petroleum and Minerals, Dhahran, 31261 Saudi Arabia; 4 Department of Pharmaceutics, College of Pharmacy, King Saud University, Riyadh, Saudi Arabia; 5 Electron Microscopy Unit, Institute for Research & Medical Consultations, Imam Abdulrahman Bin Faisal University, P.O. Box 1982, Dammam, 31441 Saudi Arabia

## Abstract

Chemotherapy, the most commonly used therapeutic method for cancer, has the inherent constraint of low bioavailability. A number of physical cancer therapeutic treatments like radiation, ultrasound, photo-acoustic/photo thermal, microwave therapies are based on locating the afflicted sites with the help of imaging, but the serious drawbacks of these treatment options are that they damage the neighboring normal tissues and/or induce undesired cancer metastasis. In addition, these methods of treatment are very expensive and not in the reach of a common man especially in the developing countries. Therefore, innovative, less invasive and cost effective treatment methods with the help of less toxic drugs have been sought for treating cancer. In this work, photo-catalytic method of killing cancer cells, using the nanostructured silver loaded tungsten oxide (Ag/WO_3_) as photo-catalysts, in conjunction with broadband UV radiation is presented. Ag/WO_3_with two different mass ratios of Ag and WO_3_ (1% Ag/WO_3_ and 3% Ag/WO_3_) were synthesized, characterized and these nanostructured materials served as photo-catalysts in the process of killing cancer cells by photo-catalytic method. The advantage of loading Ag in WO_3_ is quite evident from the observed increase in the photo-catalytic killing of the HeLa cells. This photo-catalytic enhancement was effectively caused by the development of Schottky junction between Ag in WO_3_, which led to a substantial inhibition of photo-generated charge recombination and also by the stimulation of surface plasmon resonance in silver nanoparticles, which led to the enhanced visible light absorption by the material.

## Introduction

The cancer is a group of diseases characterized by the uncontrolled growth and spread of abnormal cells^1^, and in most of the cases develop into malignant masses of tissues called tumors, and it is the leading causes of mortality and a major public health challenge worldwide. In normal body, genes in the cell nucleus, containing long strings of DNA (deoxyribonucleic acid) regulate the controlled division and function of cells and any damage to DNA causes the mutation of genes, which in turn triggers the uncontrolled division of abnormal cells, leading to the damage of vital organs. Cancer cells can detach from the original mass of tumor and migrate to new locations through blood and lymphatic system and also cancer cells produce enzymes that are capable of breaking the normal cells. For cancer diagnostics, the conventional histopathological and radiological examinations are still used for evaluating the clinical and pathologic staging, needed for cancer treatments^2^. Depends on the stage of cancer development, different treatment options like chemotherapy, radiation therapy, stem cell transplant, immunotherapy, hormone therapy, targeted drug therapy and surgery are advised^3^. The major disadvantages of the available advanced treatment options include non localized invasion to other body parts, intolerable cytotoxicity, unsystematic distribution of antitumor agents, immune to chemical agents, low bioavailability and limited option to evaluate the tumor cell response to therapies^4,5^. In spite of the drawbacks of these advanced treatment options, cancer is curable if it is diagnosed at an early stage.

Phototherapy has been used for the treatment of jaundice, cancer, dermatological conditions, and ophthalmological disorders by simply using the light of certain selected wavelength. Photodynamic therapy, on the other hand is a method of photosensitizing the action of drugs to kill cancer cells, but the major drawback of this treatment is that most of the drugs used for photodynamic therapy remain activated for a long time, leading to overdose to damage non cancer cells. In the photo-catalytic process, no drug is used, instead the nontoxic semiconductor photo-catalyst like WO_3_ generates electron hole pairs, when it is exposed to the light of appropriate wavelength and these photo-generated charge carriers mediate oxidation and reduction reactions in the cancer cell to eliminate them. The major technical limitations in the semiconductor photo-catalyst used in this process are the early recombination of charge carriers generated by photo-excitation, before they are put into proper use and the limited light absorption. The successful photo-catalyst is the one, in which the photo-generated charge carriers remain separated so that they can initiate the redox reaction to kill cancer cell or in general, to carry out any chemical reaction.

Tungsten trioxide (WO_3_) is a transition metal oxide semiconductor, with a band gap energy ranging between 2.4 eV and 3.0 eV at room temperature^6^ and hence, WO_3_ has a strong visible spectral light adsorption^7^, contrary to other photo-catalyst like TiO_2_, which has light absorption in the harmful UV spectral region due to its inherent band gap energy. In addition to the favorable region of light absorption, WO_3_ is well known for its resistance to photo-corrosion, and stable physicochemical properties^8–11^. However, there are some key factors like rapid charge recombination, low visible light absorption that limit the effective use of WO_3_ as a photo-catalyst^12^. In order to surmount these limitations of WO_3_ as a photo-catalyst, it was doped or modified with many metallic dopant like Pt, Au, Ag, and Pd and significant enhancement of photocatalytic process was observed^13,14^. Silver nanoparticles have gained increasing interest in different fields of nanotechnology and particularly in nano-medicine due to its therapeutic potential in treating a large variety of diseases. Also doping of silver in WO_3_ can favorably alter many optical and structural properties of WO_3_ and also enhances the photo-generated charge separation to promote the photo-catalytic performance^15,16^. When the light of appropriate wavelength falls on the photo-catalyst, the electron-hole pair is formed as the electrons move from the valance band to the conduction band. In aqueous environment, the photo-generated holes in the valance band oxidaize the water molecule to produce hydroxyl radicals (^•^OH) and hydroperoxyl radicals (OH_2_^•^), while, the electrons reduce the oxygen to produce a superoxide anion (O_2_^•−^) or hydrogen peroxide (H_2_O_2_)^15^. These highly reactive oxygen species (ROS) reacts with the cancer cells to terminate them by programed cell death (apoptosis) and/or unplanned cell death (necrosis) as a result of oxidative stress. ROS reacts with the cell membrane and cell interior and affects DNA, cell rigidity and surface structure leading to the killing of tumor cells and these actions can be controlled by localizing the positions of the photo-catalyst at the time of light irradiation^16–18^.

Besides, using metal-oxide nanomaterials as photo-catalysts, recently, materials such as zinc oxide (ZnO), titanium dioxide (TiO_2_), copper oxide (CuO), silicon dioxide (SiO_2_), iron oxide (Fe_2_O_3_/Fe_3_O_4_), cerium oxide (CeO_2_) have been used for other biological applications (anticancer and antitumor)^19–22^. Among all these metal-oxide nanomaterials, ZnO and TiO_2_ nanoparticles have been widely utilized as anticancer agents, owing to their positive feature like low-cost, biocompatibility, easy synthesis and enhanced cytotoxicity^23–25^.

In this work, silver loaded tungsten oxide with two different mass ratios of silver in WO_3_ (3% Ag/WO_3_ and 3% Ag/WO_3_) were synthesized and these nanostructured semiconductor materials served as photo-catalysts in conjunction with the broadband UV radiation to bring about the enhanced photo-catalytic killing of HeLa cells. It was observed that the anchoring of Ag on WO_3_ helped in the enhancement of the photo-catalytic killing of cancer cells, compared to pure WO_3_ under the same UV radiation and this photo-catalytic enhancement is further improved with the increase of Ag concentration in WO_3_ from 1% to 3%. The enhanced photo-catalytic property of Ag/WO_3_ was rationalized by studying the morphological and optical characterization by FE-SEM, TEM, XPS, and diffused reflectance. The loading of Ag on WO_3_ is quite obvious with the average particle size of WO_3_ to be 55 nm and Ag particles are much smaller with the size range of 5–10 nm. The anchoring of Ag on WO_3_ decreases the recombination of the charge carriers generated by photo-excitation through the formation of metal- semiconductor junction (Schottky junction). It is well known that the reduction of the photo-generated charge recombination is crucial for the enhancement of the photo-catalytic activity, as more charge carriers will contribute to the redox reaction. Another advantage of loading Ag on WO_3_ in the context of photo-catalysis is that the visible light absorption in the material has enhanced and this leads to the stimulation of surface plasmon resonance, which also positively contributed for the enhancement of photo-catalytic killing of cancer cells.

## Results and Discussion

### Morphology of nanostructuredWO_3_ and Ag/WO_3_

**Figure 1:**
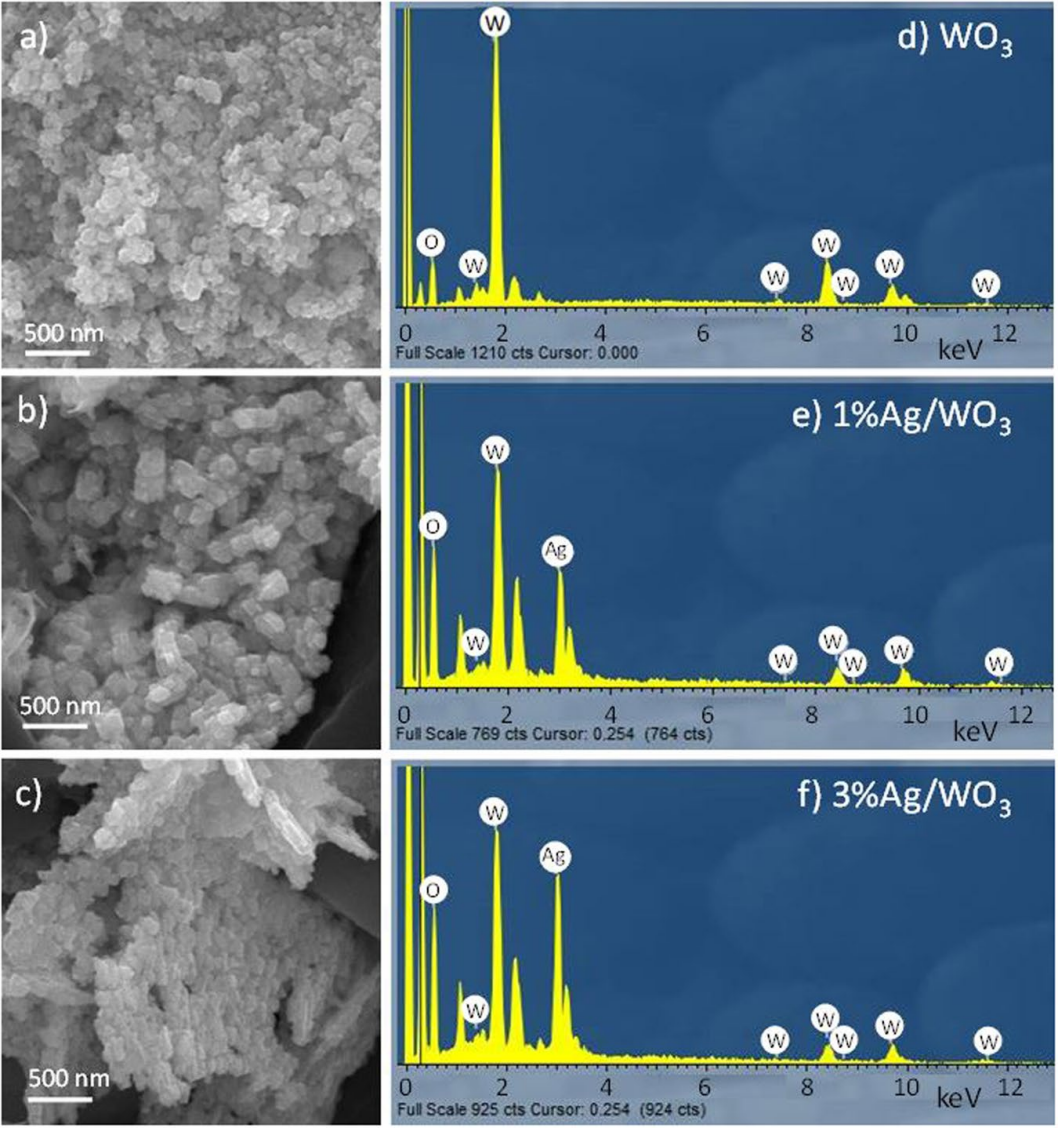
SEM images of (**a**) WO_3_, (**b**) 1% Ag/WO_3_ and (**c**) 3% Ag/WO_3_ with corresponding EDX plots (**d–f**).

The morphology of nanostructured Ag loaded WO_3_ and the pure WO_3_, synthesized in our lab were studied using field emission scanning electron microscopy (FE-SEM) and the results are shown in Fig. 1. From the image in Fig. 1a, it is quite clear that WO_3_ particles are well distributed and preserve their typical identity and the size of the particles are obviously smaller than the scale given in the legend of the electronic image. After Ag loading, the WO_3_ showed some spatial separation as few gaps are noticed in the image in Fig. 1b and the same trend is observed for 3% Ag loading on WO_3_, although a bit of particle aggregation is observed at certain places in Fig. 1c.

The chemical composition of the synthesized material was studied using FESEM-EDX. The results of different samples are depicted in Fig. 1(d–f). By Fig. 1d of pure WO_3_ sample, the presence of tungsten (W) and oxygen (O) was confirmed. Silver (Ag) loaded WO_3_ sample exhibited an additional peak at around 3 keV, which identifies the Ag peak and confirms the presence of Ag in W and O (Fig. 1e). Interestingly, the height of this Ag peak was increased on loading of 3% Ag in WO_3_ (Fig. 1f). However, the Ag signal was stronger with 3% Ag sample than 1% Ag sample probably due to use of higher Ag concentration. EDX analyses showed the existence Ag in both 1% Ag/WO_3_ and 3% Ag/WO_3_ specimens.

**Figure 2:**
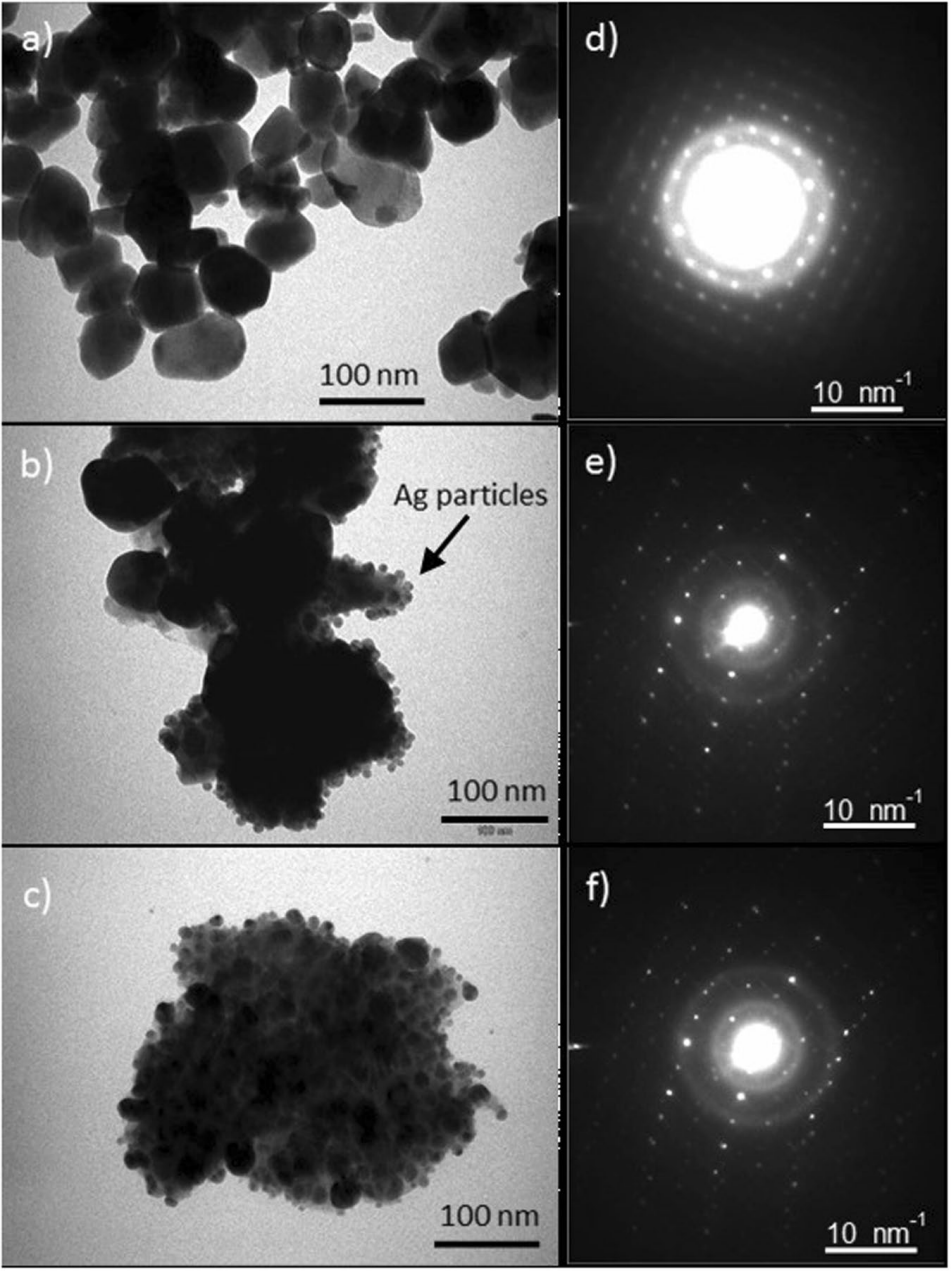
TEM images (**a**) WO_3_, (**b**) 1% Ag/WO_3_ and (**c**) 3% Ag/WO_3_ with SAD patterns. All scale bars in TEM images corresponding to 100 nm and SAED pattern are 10 1/nm.

As SEM has the inherent limitation of the image resolution to display the shape and size of the nanosized materials. Therefore, in order to understand the shape and the size of the Ag and WO_3_ particles in detail, TEM was performed on all the three synthesized specimens. The results of TEM are depicted in Fig. 2. As it can be observed in Fig. 2a, WO_3_ particles exhibit spherical shape with the diameter well below 100 nm and the estimated average particle size of WO_3_ is about 55 ± 12 nm. On the other hand, in the images for Ag loaded WO_3_ samples in Fig. 2b,c, it is quite clear that the size of the Ag particles is much smaller than WO_3_ particles with size range 5–10 nm. The loading of Ag on WO_3_ is quite obvious as Ag particles can be seen attached to the WO_3_ particles in Fig. 2b. Also, the increased concentration of Ag particles in WO_3_ is quite obvious in Fig. 2c. Selected area electron diffraction (SAED) was performed to further highlight the existence of Ag in WO_3_ by comparing the diffraction patterns of Ag loaded specimens with pure WO_3_ product. The results of SAED patterns of all the samples are presented in Fig. 2d–f. The electron patterns of Ag loaded WO_3_ specimens, 1% Ag and 3% Ag clearly showed the combination of spots as observed for pure WO_3_ specimen, confirming the presence of silver particles in tungsten oxide.

**Figure 3:**
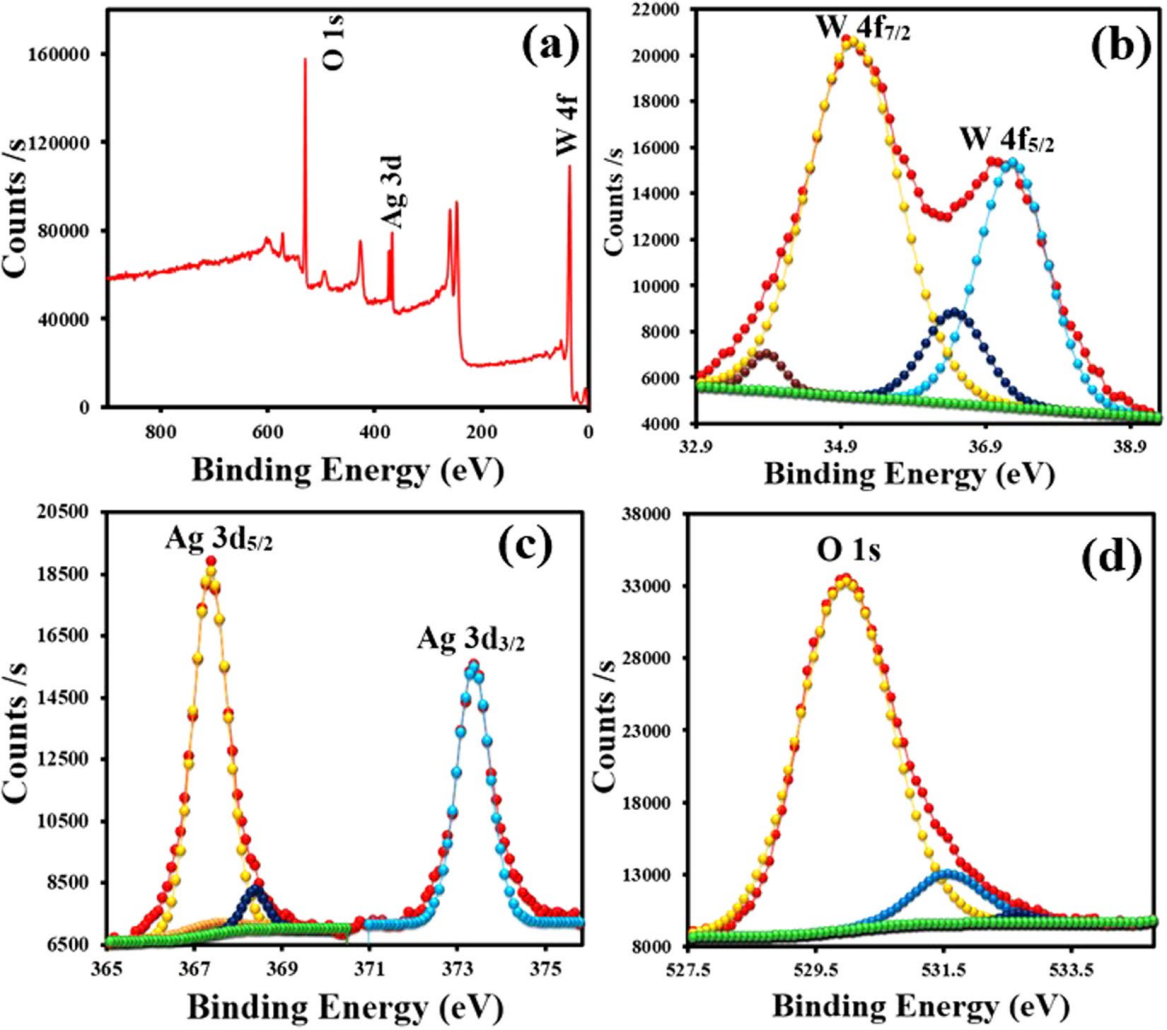
XPS Survey scan of 3% Ag/WO_3_ (**a**). High-resolution scan of W 4f (**b**), Ag 3d (**c**) and O 1s (**d**) for 3% Ag/WO_3_ surface.

The elemental characterization of 3% Ag/WO_3_ was carried out by XPS and the spectra are depicted in Fig. 3. The XPS survey scan in Fig. 3a clearly shows the presence of W(4 f), Ag(3d), and O(1s) peaks and the deconvolution of these peaks are respectively shown in Fig. 3b–d respectively. The doublet of W (4f) comprises of W (4f_7/2_) at 35.6 eV and W (4f_5/2_) at 37.8 eV with the intensity ratio of 4:3 as predicted by the spin orbit splitting of W (4 f) levels. We can notice that the observed binding energies of W (4f_7/2_) and W (4f_5/2_) in WO_3_ are higher than that of the same in metallic tungsten. This is because the six valance electrons of W are utilized for the bond formation with oxygen and the wave functions of these electrons are not spread around in the vicinity of W atom and hence the remaining electrons including the electrons in 4f levels are more strongly bound to the nucleus than that of the pure metallic tungsten atom. The XPS spectra of Ag(3d) in Fig. 3b shows a spin-orbit component Ag(3d_3/2_) at 373.8 eV and Ag(3d_5/2_) at 367.3 eV and these binding energies are apparently slightly less than that for the Ag metal for which 3d_3/2_ and 3d_5/2_ peaks are respectively at 374.3 eV and 368.3 eV. This shift could be due to the transfer of electrons from the metal to semiconductor through Schottky junction, formed in the metal semiconductor interface. Also Fig. 3c shows the O (1s) peak of WO_3_.

### Optical characterization of nano photo-catalysts

**Figure 4:**
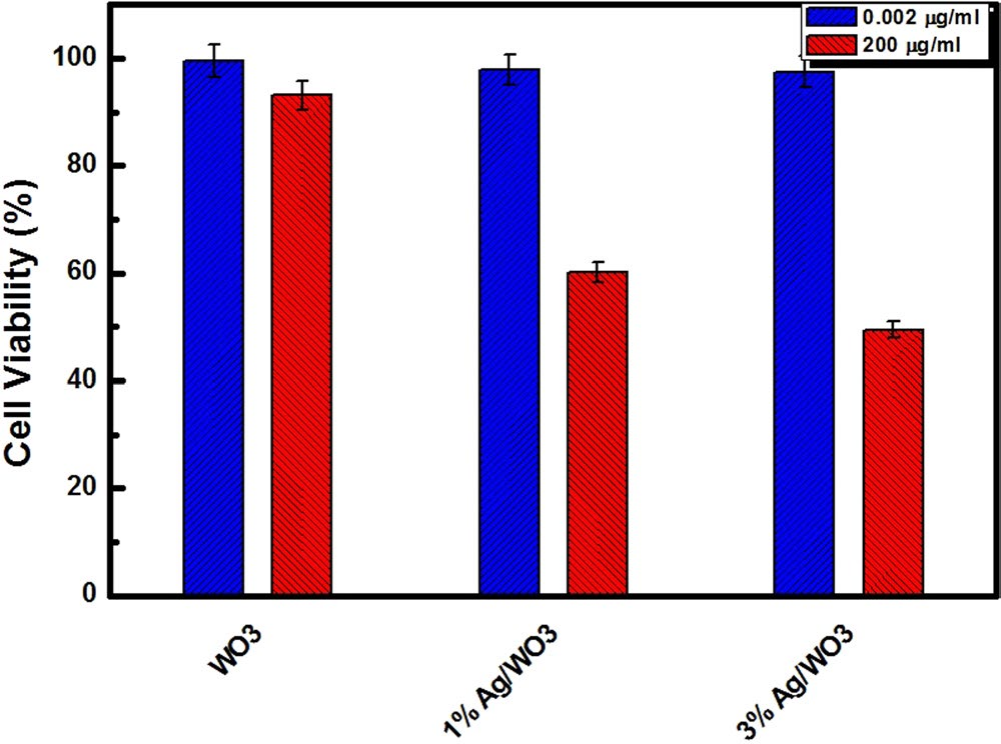
(**a**) UV–VIS diffuse reflectance spectra of pure WO_3_, 1% Ag/WO_3_ and 3% Ag/WO_3_. (**b**) Tauc plot of pure WO_3_, 1% Ag/WO_3_ and 3% Ag/WO_3_.

Optical characterization of the synthesized material was carried out to study the effect of silver doping on WO_3_ in terms of light absorption and the change of the band gap energy. Figure 4a shows the UV–VIS diffuse reflectance spectra of pure WO_3_, 1% Ag/WO_3_ and 3% Ag/WO_3_, where it is quite clear that as the concentration of Ag in Ag/WO_3_ becomes higher, the reflectance of the material at the higher wavelength decreases, indicating more absorption of light in this spectral region. The enhancement of absorption in this region can be attributed to the surface plasmon resonance (SPR), brought about by nano silver particles in WO_3_. The surface plasmon resonance is due to the collective oscillation of the conduction electrons in the metal nanoparticles in the presence of the electric field of the incident light radiation and this effect enhances the light absorption in the appropriate spectral region. The effect of Ag loading on WO_3_ in the band gap of the modified material is depicted in Fig. 4b by using Tauc plot^11^, which is basically (*Fhv*)^2^ versus photon energy (*hv*) for direct band gap material, where *F* is the Kubelka–Munk function^11^, which is the equivalent of absorption coefficient deduced from reflectance as in equation 1 and *hv* is the incident photon energy. Generally the absorption coefficient (*α*) is related to the band gap energy (E_g_) as shown in equation 2, where A is a constant known as band tailing parameter and n is the power factor of the transition mode which depends on the nature of the material. The value of n in equation 2 is taken as ½ for direct band gap materials and taken as 2 for indirect band gap materials, where the transitions are assisted by phonons to conserve momentum. Transforming equation 2 into linear form leads to equation 3 and extrapolating the linear part of the Tauc plot and its intercept on the x axis directly yields the band gap energy of the material.

1 $$\alpha =F(R)=\frac{{(1-R)}^{2}}{2R}$$

2 $$\alpha ={\frac{A(hv-{E}_{g})}{hv}}^{\frac{1}{2}}$$

3 $${(\alpha hv)}^{2}=A(hv-{E}_{g})$$

The band gap energy *E*_*g*_ of WO_3_ estimated from Fig. 4b is 2.88 eV, which is in good agreement with the previously reported value^26^ and with the loading of Ag in WO_3_ the band gap energies reduced to 2.81 and 2.65 eV respectively for 1% Ag/WO_3_, and 3% Ag/WO_3_. The narrowing of the band gap energy of a semiconductor material in the presence of any electron donating or electron accepting foreign atom is quite natural as the new donar level or acceptor level are formed respectively near the conduction band and the valance band of the semiconducting material. As the loading of the foreign atom increases, the density of states of these atoms increase and form an energy band like continuum of states that causes the shrinking of the band gap energies. In our case, silver has one valance electron and hence, when it is loaded to a semiconducting material, the new energy levels are formed near the valance band and as mentioned earlier, the density of the states of these levels increases with the loading concentration of silver and this accounts for the reduction of band gap energies with increased Ag loading on WO_3_.

### Photocatalytic killing of HeLa cells

**Figure 5:**
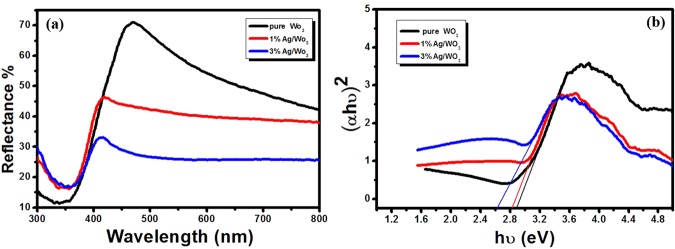
The survival viability of Hela cells in the dark in the presence of different photo-catalysts. Blue bars for low concentrations and red bars for higher concentrations of photo-catalysts.

The cytotoxicity of dark cultured nanoparticles to HeLa cells is shown in Fig. 5, where we can notice that the viability of HeLa cells decreases with the increased concentrations of the photo-catalyst used in the process. However, from Fig. 5, it is observed that with the higher concentrations of catalyst (200 μg/ml), interacting with HeLa cells for 24 hours in dark, the survival rate of HeLa cell with pure WO_3_, 1% Ag/WO_3_, and 3% Ag/WO_3_ are 93%, 60%, 49.5% respectively. Also the survival rate of HeLa cells as high as 90% was observed even with very low concentration of catalysts (0.002 μg/ml) interacting with HeLa cells for 24 hours. Accordingly, in the absence of light, WO_3_ and Ag/WO_3_ are found to be nontoxic, which is in agreement with the literature^27^.

**Figure 6:**
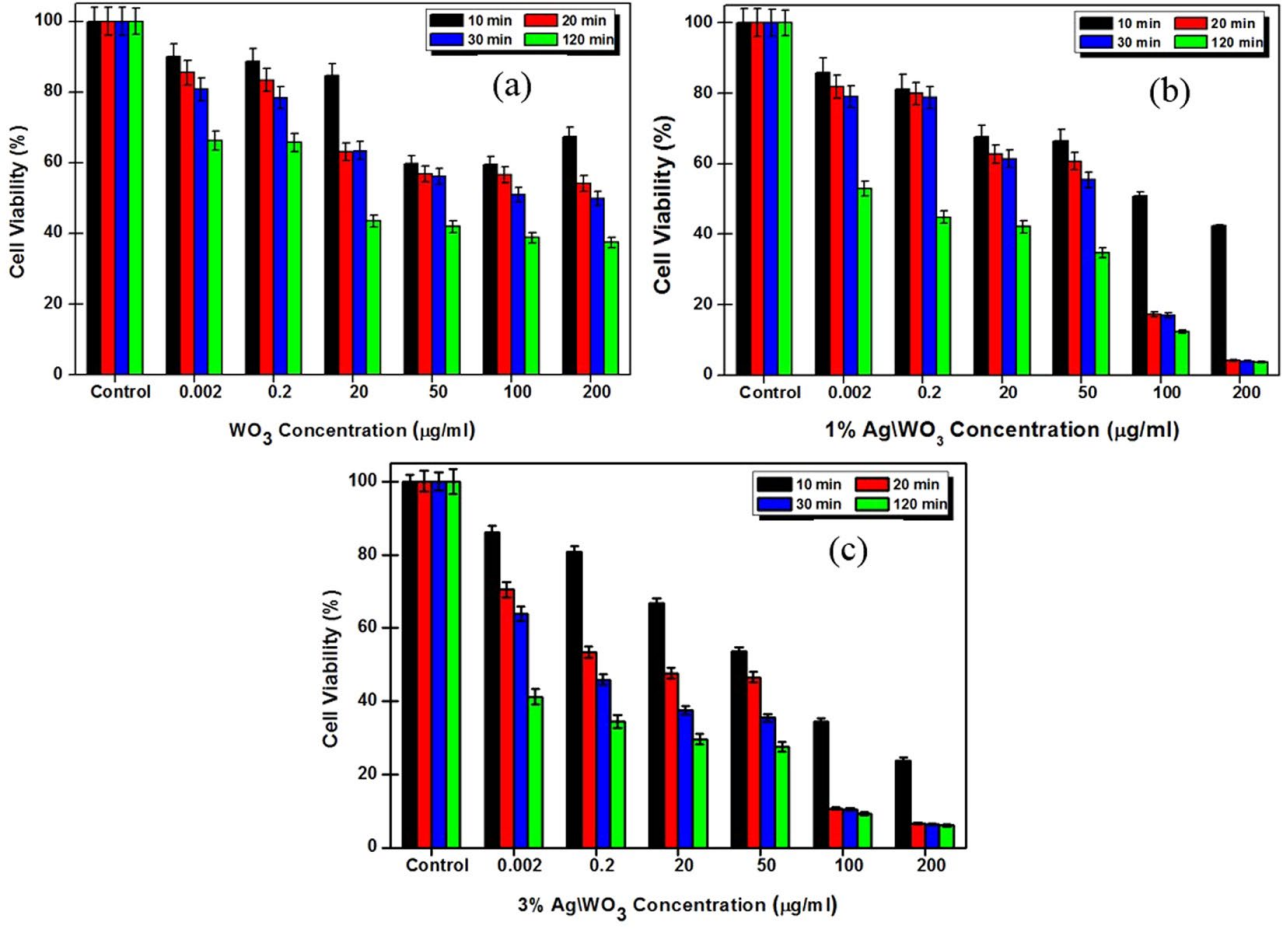
Alamar blue cytotoxicity assay of HeLa cell line *in vitro* treated with different concentration of (**a**) WO_3_, (**b**) 1% Ag/WO_3_, and (**c**) 3% Ag/WO_3_ at different UV irradiation time. Each column shows the mean values of three different experiments.

The effect of UV irradiation on WO_3_, 1% Ag/WO_3_, and 3% Ag/WO_3_ in the process of photo-catalytic killing HeLa cancer cells at different irradiation times (10, 20, 30 and 120 min) is presented in Fig. 6. The result indicates that in order to achieve 50% cell viability, 100 μg/ml of 1% Ag/WO_3_ has to be irradiated for 10 minutes (Fig. 6b), whereas, the same cell viability is obtained only with 50 μg/ml of 3% Ag/WO_3_ in the same irradiation time (Fig. 6b). In the case of pure WO_3_, it is observed that only 100 μg/ml of photo-catalyst with the extended irradiation time of 30 minutes could yield the same 50% cell viability (Fig. 6a). It is also clear from Fig. 6c that in order to achieve 90% killing of the HeLa cells by photo-catalytic process, 100 μg/ml of 3% Ag/WO_3_ needs to be irradiated for 20 minutes, whereas 1% Ag/WO_3_ cannot reach 90% of killing with this concentration and duration. Hence it is quite clear from our results that, in order to achieve a required level of killing of HeLa cells with particular photo-catalyst, there is a trade-off between the concentration of the catalysts and the duration of irradiation time. Our result shows that among the three photo-catalysts tried in this work, 3% Ag/WO_3_ is the most effective one for the killing of cancer cells and this can be attributed to the increased level of light absorption and charge separation with the increased Ag content in WO_3_, which makes a larger number of charge carriers available for the photo-catalytic reaction to produce more ROS.

**Figure 7:**
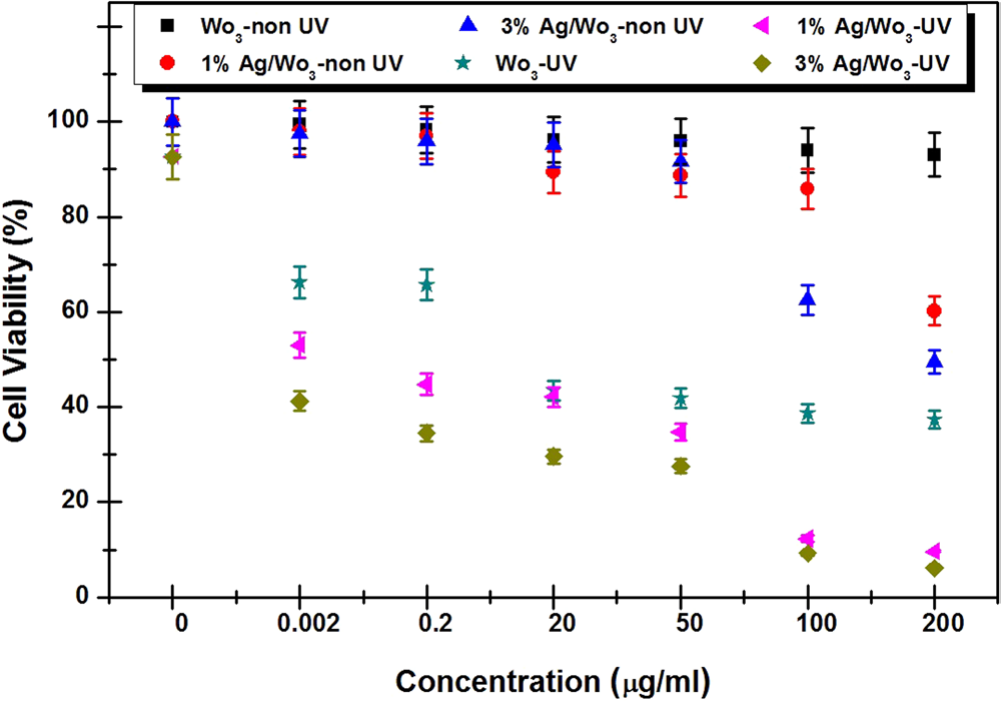
Survival viability of the HeLa cells under different experimental conditions.

The parametric (duration and concentration) dependence of cancer cell viability in the absence of light (dark) and under UV irradiation is summarized in Fig. 7. The 3% Ag/WO_3_ photo-catalyst under dark shows no cytotoxicity until the concentration reaches as high as 50 μg/ml, and reaches to the cell viability of 50%, when the catalytic concentration was 200 μg/ml. On the other hand, the highest killing of cancer cells was achieved under the UV irradiation with the same catalysts and the same catalytic concentration. It was also noticed that the effect of UV radiation (without catalyst) is minimal with the observed cell survival viability of 92% even after 2 hours of irradiation. So it is quite evident that, in the presence of light and catalyst, the photo-catalytic process is triggered and this leads to the reduction in the cell survival viability with increased concentration. The best HeLa cell viability observed in this work is 38% for pure WO_3_, 9% for 1% Ag/WO_3_ and close to 0% for 3% Ag/WO_3_. The increased photo-catalytic killing with higher concentration of catalyst is due to the availability of more active sites and the increased killing with higher Ag content is due to the enhanced light absorption and reduced charge recombination brought about by the presence of Ag in WO_3_^27^. For striking a balance, 3% of Ag loaded WO_3_ with low concentration can be used effectively for the treatment for cancers under UV light. This possible modality can easily be used for the treatment of tumors such in oral cavity, trachea, skin, and urinary bladder under UV light, which has very low penetration depth in human tissue.

**Figure 8:**
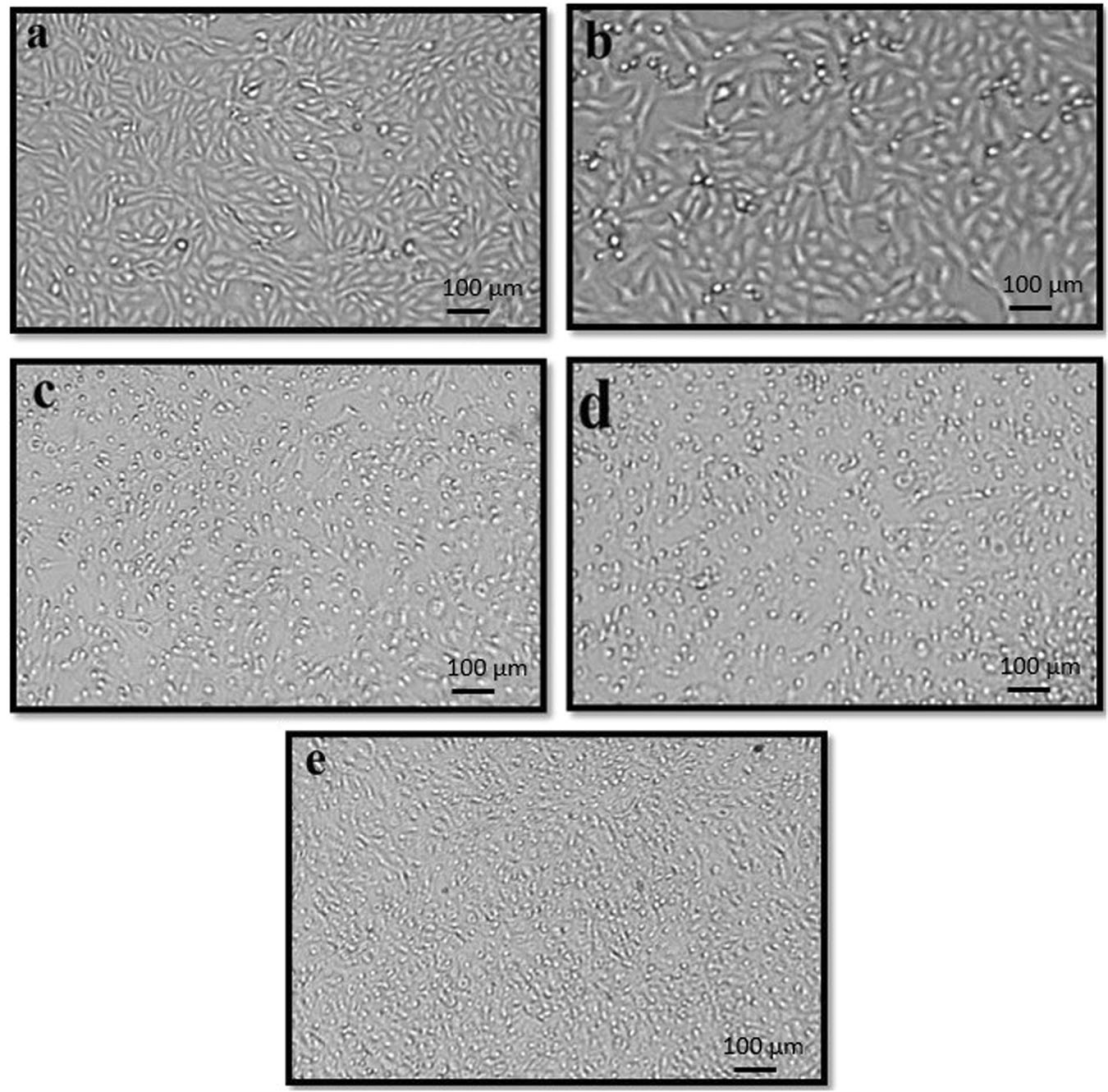
Inverted microscopic images of HeLa cells (Magnification 40 × 0.6). (**a**) With no nanoparticles or light. (**b**) After 2 hours of light irradiation without the nanoparticles. (**c**–**e**) After 2 hours of light irradiation with the 200 μg/ml WO_3_, 1% Ag/WO_3_, 3% Ag/WO_3_ nanoparticles, respectively.

Figure 8 shows microscopic images of HeLa cells before treatment (Fig. 8a), after 2 hours of UV irradiation without photo-catalysts (Fig. 8b), and after two hours of photo-catalytic reaction with the 200 μg/ml of WO_3_, 1% Ag/WO_3_, 3% Ag/WO_3_ nanoparticles (Fig. 8c–e respectively). It is quite clear from Fig. 8b that after 2 hours of UV irradiation, an insignificantly small difference is noticed between Fig. 8a and b (only 8% killing after 2 h irradiation). Also a careful examination of Fig. 8c–e, we can notice a gradual increase of the killing of HeLa cancer cells with the increased content of Ag in WO_3_ and very emphatic difference can be noticed with respect to Fig. 8a and b. These visual images also substantiate that the enhanced killing of HeLa cell is only due to the photo-catalytic process and not by simple UV light irradiation.

### Mechanism for photocatalytic killing of cancer (HeLa) cells

**Figure 9:**
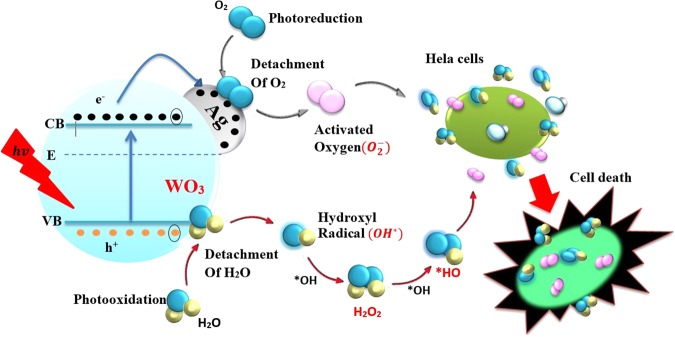
Schematic sketch of the proposed mechanism for the photocatalytic killing of cancer (HeLa) cells.

The proposed scheme of photo-catalytic mechanism that leads to the killing of cancer cells using Ag/WO_3_ as a photo-catalyst is depicted in Fig. 9. In the case of pure WO_3_ as a photo-catalyst, the electron hole pairs are generated, when electrons in valance band absorb the photons that have energy equal to or higher than the band gab energy of WO_3_ (2.89 eV). These photo-generated charge carriers are deactivated through two possible processes. The first process is the preferred one in which, the photo-generated charges mediate redox reaction, leading to the oxidation of H_2_O to form OH radicals as in equations 4 to 6 and the reduction of H_2_O and O_2_ to form reactive oxygen species (ROS) such as singlet oxygen, hydroxyl radical, superoxide, hydroperoxyl radical, and hydrogen peroxide as in equations 7 to 9 which are responsible for the oxidative stress in cellular systems to induce cellular death by apoptosis or necrosis. In the second deactivation process, the generated electron hole pairs radiatively or non-radiatively recombine (equation 10), instead of involving in the redox reaction, which weakens the photo-catalysis. In order to minimize this undesired photo-generated charge recombination, usually the semiconductor photo-catalyst is either combined with another appropriate semiconductor to form a heterojunction or loaded with a noble metal like Ag to form a Schottky junction to trap the charges and thereby to minimize the recombination process. In our case n- type WO_3_ is loaded with Ag and in this case, the work function of WO_3_ (5.7 eV), which is the energy separation between the vacuum level and the Fermi level is larger than the work function of silver (4.7 eV). Hence the electron-hole pairs generated by the photo-excitation migrate from silver to the conduction band of WO_3_, creating excess of electrons in the semiconductor side and holes in the metal side of the Schottky junction. Also due to this charge migration, there is a development of a new potential profile in the metal semiconductor junction, leading to the band bending. This inflexed band inhibits the electron hole recombination in the space charge region and make these charge carriers available for effective photo-catalytic process through redox reaction, to generate ROS (OH^•^, HO_2_^•^, O_2_^•−^, H_2_O_2_) and this leads to oxidative stress in cellular systems to induce cellular death by apoptosis or necrosis.

Oxidation process:

4 $${h}^{+}+{O}_{2}^{\bullet -}\to {}^{1}O_{2}$$

5 $${h}^{+}+{H}_{2}O\to {H}^{+}+{}^{\bullet }OH$$

6 $${h}^{+}+O{H}^{-}\to {}^{\bullet }OH$$

Reduction process:

7 $${e}^{-}+{O}_{2}\to {O}_{2}^{\bullet -}+{H}^{+}\to H{O}_{2}^{\bullet }\mathop{\to }\limits^{H{O}_{2}^{\bullet }}{H}_{2}{O}_{2}+{O}_{2}$$

8 $${e}^{-}+{H}_{2}{O}_{2}\to O{H}^{\bullet }+O{H}^{-}$$

9 $${e}^{-}+{O}_{2}\to {O}_{2}^{\bullet -}\to O{H}^{\bullet }+O{H}^{-}+{O}_{2}$$

10 $$W{O}_{3}+hv\to W{O}_{3}({h}_{vb}^{+};{e}_{cb}^{-})\to recombination$$

## Conclusions

We synthesized, nanostructured tungsten oxide (WO_3_) and different percentages (1% Ag/WO_3_ and 3% Ag/WO_3_) of silver loaded WO_3_ and applied these materials for the photo-catalytic killing of HeLa cancer cells cultured *in vitro* under UV radiation of 365 nm wavelength. The results clearly showed that 3% silver loaded WO_3_ has the best photo-catalytic efficiency to kill the HeLa cancer cells. Therefore, Ag/WO_3_ could be very well applicable for *in-vitro* and malignant tumors treatment, by directly injecting into the malignant tumorous tissues, which results in the concurrent high therapeutic efficacy and negligible damages to the neighboring normal tissues and/or organs. It is worth mentioning that WO_3_ is highly stable, inert as well as biocompatible material with very little side effects to human body. The increased photo-catalytic killing of HeLa cancer cell is explained in the light of the improved morphological and optical properties of Ag/WO_3_, which is also the part of the study of this work.

## Experimental

### Synthesis of nano photo-catalysts

For the synthesis of pure WO_3_ nanoparticles, the aqueous solution of tungsten salt was initially made by dissolving 8.24 g of Na_2_WO_4_. 2H_2_O in 50 ml of deionized (DI) water at 25 °C under continuous stirring. 5 ml HCl was added to the tungstate solution and subjected this solution for further stirring (400 rpm) at 25 °C for 2 hours, until the precursor became dense and yellow in color. The precipitate, thus obtained was washed in DI water, dried at a temperature of 120 °C in an oven for 15 hours, further calcined in a programmable furnace at 400 °C for 4 hours, and ground to get the final material in powder form. In the process of synthesizing 1% Ag/WO_3_ and 3% Ag/WO_3_ nano photo-catalysts, an appropriate concentrations of aqueous silver nitrate solution was mixed with 1 gram of fine WO_3_ nano-powder, and this paste was mixed thoroughly dried and calcined at 400 °C for 4 hours to get Ag/WO_3_.

### Characterization

For the morphological characterization of the synthesized photo-catalysts, Transmission electron microscope (TEM, FEI, Morgagni, Czec Republic) and Field emission scanning electron microscope (FE-SEM, TESCAN FERA3) were used. The operating voltages of FE-SEM and TEM were 20 kV and 80 kV respectively. For TEM analysis, the particle dispersion was dropped onto the carbon-coated Cu grids and was air-dried before mounting on the microscope and Gatan digital micrograph software was used to estimate the particle sizes from electronic images. Quantitative elemental analysis was carried out by Energy Dispersive X-ray (EDX) equipment Apollox SDD (silicon Drift Detector) attached to the FE-SEM instrument. For optical characterization, Spectrophotometer (JASCO, V-570), equipped with diffused reflectance option with the help of integrated sphere was used to get the reflectance spectra of the synthesized materials.

### Growth media and preparation of nano catalyst stock solutions

Dulbecco’s Modified Eagle’s medium (DMEM, Sigma-Aldrich, Germany) in 500 ml container was supplemented with 10% (50 ml) of Fetal Bovine Serum (FBS, Sigma -Aldrich, Germany) and 1% (5 ml) of antibiotic-antimycotic (Sigma -Aldrich, Germany).

The stock solutions of 1 mg/ml concentration of WO_3_, 1% Ag/WO_3_, or 3% Ag/WO_3_ were prepared by adding fine powder into sterilized DI water and sonicated for 1 to 5 min to reach the maximum solubility. The prepared stock solution of the photo-catalyst and the supplemented DMEM were dispensed in right amount to prepare different concentrations (200, 100, 50, 20, 0.2, and 0.002 μg/ml) of nanoparticle solutions through serial dilution.

### Cell culturing and Cytotoxic assay

HeLa cells are human cervical cancer cell lines, which was acquired from King Faisal Specialist Hospital and Research Center (KFSHRC- Riyadh, SA). T 75 cm^2^ tissue culture flasks (Vented) were used for cell culturing and were incubated at 37 °C in 5% CO_2_ environment. 1 ml of Trypsin–EDTA (0.05%) phenol red (Sigma –Aldrich, Germany) was used as an agent for the splitting of cells and then seeded the cells in new flasks or treatment wells.

The cytotoxicity of the cancer cells was studied with i) WO_3_, 1% Ag/WO_3_, and 3% Ag/WO_3_ nanoparticles without irradiation, ii) WO_3_, 1% Ag/WO_3_, or 3% Ag/WO_3_ nanoparticles in conjunction with UV irradiation and iii) UV irradiation without photo-catalysts. The presented results are the average value of three independent measurements. At first 400 μl of Hela cells with a cell density of 3 × 10^5^ cells/ml was placed in 24-well plate and incubated for 48 hours at 37 °C in 5% CO_2_ environment. The protocol used to estimate the effect of nanoparticles was aspirating the media and adding the different concentrations of nanoparticles solutions. The cells were incubated for 24 hours, and subsequently 40 μl alamar blue reagent (Bio-Rad, UK) was added in each well and again re-incubated for another 2–3 hours. For the quantification of the cell survival, the fluorescence emission intensity of the reagent was measured by using a microplate reader (iM3) at 560 nm excitation wavelength and 590 nm emission wavelength and comparing it with the fluorescence emission of the wells with untreated (control) cancer cells.

### Photo-catalytic studies

In order to study the photo-catalytic process using WO_3_, 1% Ag/WO_3_, or 3% Ag/WO_3_ as photo-catalysts, the cell culture growth media was mixed with different concentrations of above catalysts, re-incubated at 37 °C in 5% CO_2_ environment for 30 minutes. The samples thus prepared were subjected to UV irradiation (wavelength 365 nm and intensity 30 mW/cm^2^) for 10, 20, 30, 40, 50, and 60 min. The survival viability of Hela cells were evaluated by studying the fluorescence from alamar blue reagent.
